# Strong nutrient-plant interactions enhance the stability of ecosystems

**DOI:** 10.1038/s42003-021-02737-3

**Published:** 2021-10-20

**Authors:** Zachariah G. Schonberger, Kevin McCann, Gabriel Gellner

**Affiliations:** Department of Integrative Biology, University of Guelph, Guelph, ON N1G 2W1 Canada

**Keywords:** Ecological modelling, Ecosystem ecology, Food webs

## Abstract

Modular food web theory shows how weak energetic fluxes resulting from consumptive interactions plays a major role in stabilizing food webs in space and time. Despite the reliance on energetic fluxes, food web theory surprisingly remains poorly understood within an ecosystem context that naturally focuses on material fluxes. At the same time, while ecosystem theory has employed modular nutrient-limited ecosystem models to understand how limiting nutrients alter the structure and dynamics of food webs, ecosystem theory has overlooked the role of key ecosystem interactions and their strengths (e.g., plant-nutrient; R-N) in mediating the stability of nutrient-limited ecosystems. Here, towards integrating food web theory and ecosystem theory, we first briefly review consumer-resource interactions (C-R) highlighting the relationship between the structure of C-R interactions and the stability of food web modules. We then translate this framework to nutrient-based systems, showing that the nutrient-plant interaction behaves as a coherent extension of current modular food web theory; however, in contrast to the rule that weak C-R interactions tend to be stabilizing we show that strong nutrient-plant interactions are potent stabilizers in nutrient-limited ecosystem models.

## Abstract (web summary)

Schonberger et al. combine nutrient-plant interactions and consumer-resource interactions into simulation models, and explore how the strength of these interactions affects ecosystem stability across several types of trophic modules. Their study concludes that strong nutrient-plant interactions increase ecosystem stability, contrasting the notion that weak consumer-resource interactions tend to be stabilizing.

## Introduction

Modular theory has given ecologists tremendous insight into the stabilizing mechanisms that underlie food webs in space and time^1–3^. It is predicated on the idea that complex networks can be reduced to subnetworks (i.e., modules) so their dynamical properties can be studied in detail, and then these properties can be used to piece back together an understanding of whole web dynamics^4–6^. Importantly, different modules exist at different levels of complexity. Higher order modules include the diamond module, omnivory module, and three species food chain, while a single C–R (consumer–resource) interaction is representative of a base module. The dynamical properties of higher order food web modules have often been deduced through an understanding of the coupled C–R subsystems (i.e., base modules) that exist within them^7–10^.

Despite the utility of a modular approach, and the pioneering work on ecosystem modules over 30 years ago by Donald DeAngelis, modular theory has seldom been adopted as a means for understanding the dynamics of ecosystem modules. A nutrient-limited ecosystem model (i.e., an ecosystem module) is comprised of a limiting-nutrient pool coupled to some community assemblage, which in turn recycles nutrients back to the limiting-nutrient pool either directly or indirectly through a detrital compartment^11^. Regardless of the community assemblage, the basal interaction of the model necessarily exists between the limiting-nutrient pool (N) and an autotroph (R) (i.e., the R–N module), where the limiting-nutrient pool grows independent of its density and has dynamical properties that vary from the classic C–R module^12^. Nonetheless, the R–N base module has not yet been considered as a means for understanding the dynamical properties of nutrient-limited ecosystem models within a modular framework. R–N is a fundamental interaction of ecosystems and in light of the push for integrating population-level interactions with material cycling processes^11,13,14^, the R–N subsystem merits further consideration with respect to modulating ecosystem dynamics.

Here, we begin by revisiting the findings of modular food web theory, reviewing how an understanding of the C–R base module can allow us to understand how the interaction strength and placement of C–R subsystems within higher order modules corresponds to predictable dynamical outcomes. We then use analytical and numerical techniques to establish a generalized relationship between interaction strength and stability for the R–N module, followed by a numerical analysis to understand how the R–N module operates as a subsystem within higher order systems, with the ultimate goal of relating the structure of the R–N module to predictable dynamical outcomes. Our results allow a coherent framework for integrating food web and ecosystem theory and highlights the different role interaction strength plays for stability in a unified framework.

## Results

### Review of C–R stability theory

**Fig. 1:**
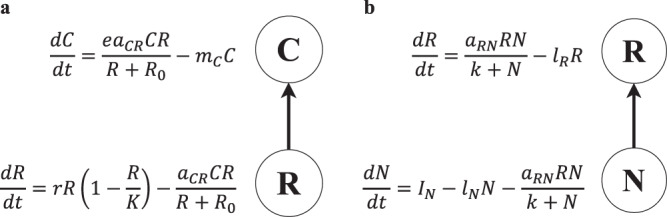
C–R and R–N base modules. **a** Rosenzweig–MacArthur C–R module modelled with Holling type II functional response and logistic resource growth, where $R$ is resource biomass and $C$ is consumer biomass. Parameters: $r$ is the intrinsic growth rate of $R$, $K$ is the carrying capacity of $R$, ${a}_{{\mathrm {CR}}}$ is the attack rate of $C$ on $R$, $e$ is the assimilation rate of $C$, ${R}_{0}$ is the half-saturation density of $C$, ${m}_{R}$ and ${m}_{C}$ are the mortality rates of $R$ and $C$, respectively. **b** R–N module modelled with a Monod nutrient uptake equation and external nutrient input, where $N$ is a limiting-nutrient pool and $R$ is the resource biomass. Parameters: ${I}_{N}$ is external nutrient input to $N$, ${a}_{{RN}}$ is nutrient uptake rate by $R$, $k$ is the half-saturation density of $R$, ${l}_{N}$ and ${l}_{R}$ are nutrient loss rates from $N$ and $R$, respectively.

**Fig. 2:**
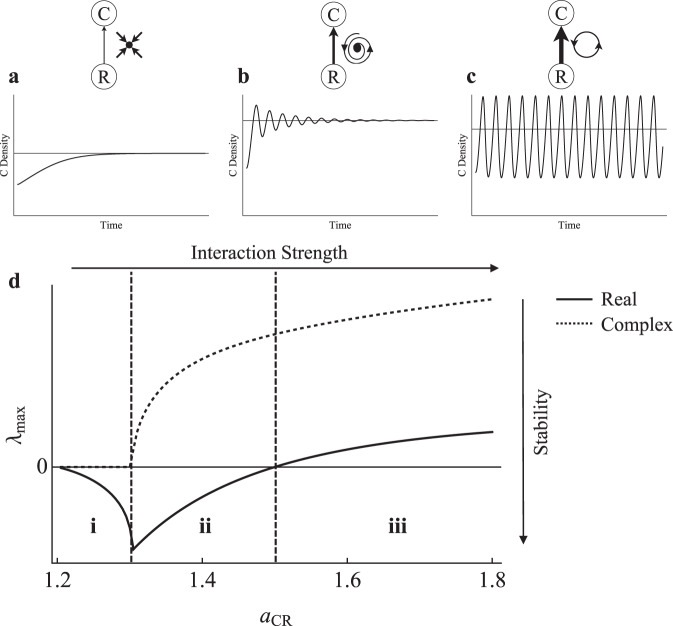
C–R checkmark stability response. **d** Local stability (real and complex parts of the dominant eigenvalue; ${\lambda }_{{\max }}$) as a function of interaction strength (${a}_{{{\mathrm {CR}}}}$) for the Rosenzweig–MacArthur C–R module. Time series reflect dynamics associated with region **i**, **ii**, and **iii**, respectively, following a perturbation that removes 50% of consumer biomass: **a** Stable equilibrium; monotonic dynamics. **b** Stable equilibrium; overshoot dynamics. **c** Unstable equilibrium; limit cycle. Boldness of arrows indicates the strength of interaction (${a}_{{CR}}$).

To set the context for how the R–N module will be used to understand the dynamics of nutrient-limited ecosystem models, we first briefly review stability results from modular food web theory. We do this by laying out a set of examples that serve to illustrate that in general, strong C–R interactions promote oscillatory dynamics while carefully placed weak C–R interactions dampen them^5^. We begin with the Rosenzweig–MacArthur C–R system as our base C–R module (Fig. 1a). It is biologically supported and produces a range of biologically plausible dynamics^5^, making it an appropriate system for this analysis. It exhibits three different dynamical phases over a gradient of interaction strengths (energetically defined sensu Nilsson et al. 2018) such that increasing the attack rate (${a}_{{CR}}$) increases interaction strength^15^ (Fig. 2). We use the return time after a small perturbation (i.e., eigenvalues) to highlight the natural stability trade-off that occurs as interaction strength is changed, (i.e., the “checkmark” stability pattern)^5,6^. Equations and parameters can be found in Supplementary Results 1A. We draw your attention to three notable dynamical phases of the C–R module. At low interaction strengths the dominant eigenvalue (${\lambda }_{{\max }}$) is negative and real and the C–R module follows a monotonic return to a stable equilibrium (Fig. 2a). During this phase ${\lambda }_{{\max }}$ decreases from 0 (i.e., where ${a}_{{{CR}}}$ allows the consumer to persist) to more negative values and thus stronger interactions tend to increase stability (Fig. 2d, i). At moderate interaction strengths, there is a sudden shift to eigenvalues with a non-zero complex part and population dynamics overshoot the equilibrium (Fig. 2b). Increases in interaction strength then further excite population dynamics and we observe less stable dynamics across this phase (Fig. 2d, ii). Last, the system reaches a Hopf bifurcation where the dominant eigenvalue becomes positive, yielding sustained cycles or oscillations (Fig. 2c, d, iii). As interaction strength increases across this phase, it is difficult to determine stability from the magnitude of a positive dominant eigenvalue; however, destabilization with increased interaction strength is readily observed in that the cycles become increasingly larger oscillations with a high coefficient of variation (CV)^5^. Note that while the Rosenzweig–MacArthur C–R system is shown here under a single set of parameters, analysis of the Jacobian shows the qualitative results to be general^5^. Moreover, the qualitative stability pattern remains for a type I and type III functional response^5^.

**Fig. 3:**
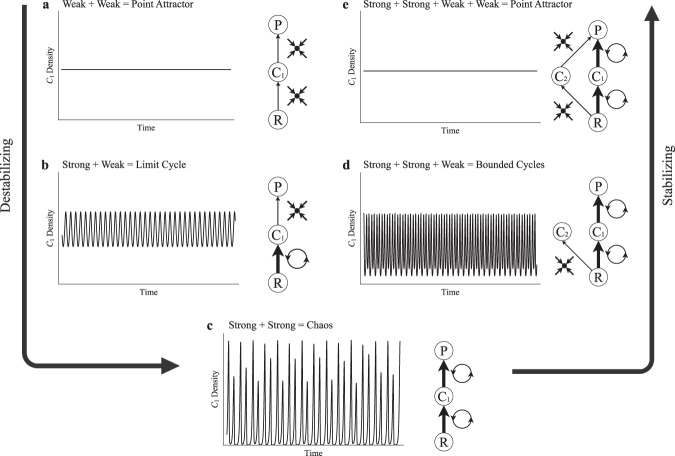
Algebra of C–R modules. Time series showing the general dynamical outcomes for the food chain and diamond module at steady state with varied combinations of C–R interaction strengths. **a** Weak–weak interaction; point attractor. **b** Strong–weak interaction; limit cycle. **c** Strong–strong interaction; chaos. **d** Strong–strong, weak interaction; limit cycle. **e** Strong–strong, weak–weak interaction; point attractor.

We now couple C–R modules into higher order food web modules to demonstrate how the addition of weak and/or strong interactions to a system can be used to predict dynamics at steady state (Fig. 3), constituting the “algebra” of C–R modules. Equations and parameters can be found in Supplementary Results 1B–D. We start with the three trophic level food chain (Fig. 3a), consisting of two coupled C–R modules (i.e., C_1_-R and P–C_1_). Theory has tended to find two weakly interacting C–R modules to generally produce locally stable equilibria^16^ (Fig. 3a). Increasing the strength of the C_1_–R interaction causes it to act like an oscillator (see Fig. 2c, above), and with enough increase this underlying oscillation is reflected in the limit cycles of the entire food chain (Fig. 3b). If the P–C_1_ interaction is strengthened as well, we end up with two coupled oscillators—the recipe for chaos^17,18^ (Fig. 3c). As such, coupled strong interactions are not surprisingly the recipe for complex and highly unstable dynamics.

Following McCann et al.^19^, we now add a weakly coupled consumer C_2_ to the food chain system of Fig. 3c. This weak consumer essentially draws energy away from the strong P–C_1_–R pathway and in doing so partially mutes the coupled oscillators, bringing the dynamics back to a more even limit cycle (Fig. 3d) and under certain conditions can drive equilibrium dynamics^19^. Last, the predator is weakly coupled to C_2_, creating a strong and weak pathway. The second weak interaction further draws energy away from the strong pathway, muting the oscillators entirely and bringing the system in this example to a point attractor (Fig. 3e). These examples show that well placed weak interactions (i.e., non-oscillatory phases, Fig. 2a, b) can be used to draw energy away from strong pathways and act as potent stabilizers of potentially oscillatory pathways. Note that weak interactions play a similarly stabilizing role in the omnivory module^20^ and further, weak interactions have been shown to stabilize large food web networks^4,6^ suggesting the principles derived from modular theory scale up to whole systems. Taken altogether, the oscillatory nature of strong C–R interactions generally promotes oscillatory dynamics in higher order systems, while the careful placement of weak C–R interactions—which are monotonic in nature—act to dampen oscillations. Although not discussed to our knowledge, we conjecture that if a subsystem exists such that strong interactions lead to monotonic dynamics (i.e., without oscillatory decay), strong interactions in this case would serve as a potent stabilizer. Below, we show the R–N module appears to be such a case.

### R–N module and stability

Towards understanding how the R–N subsystem may interact in a higher order system, we first briefly consider the stability of the R–N module alone (akin to what we discussed for the C–R module above). The R–N module consists of a resource that takes up nutrients according to a Monod-like growth term, is open to flows from the external environment as a result of geochemical processes, and nutrients are lost to the external environment according to a linear term^11^ (Fig. 1b). Performing a local stability analysis about the interior equilibrium reveals the R–N module to be locally stable for all biologically feasible parameterizations, as determined by the signs of the trace and determinant of the Jacobian matrix (see Supplementary Results 2B). We now perform further numerical and analytical analyses to understand how stability is influenced by interaction strength.

**Fig. 4:**
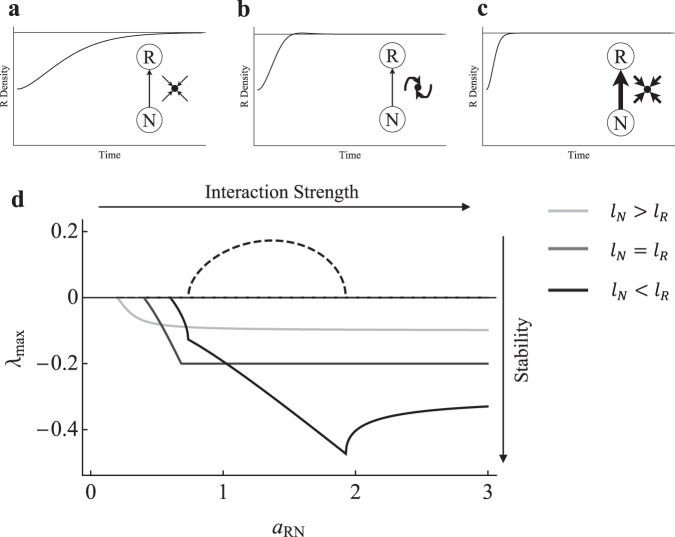
R–N stability response to increasing interaction strength. Time series showing R density following a perturbation that lowered R density to 50% of equilibrium density for **a** low (${a}_{{RN}}=0.8$), **b** intermediate (${a}_{{RN}}=1$), and **c** high maximum rate of nutrient uptake (${a}_{{RN}}=2.8$). **d** Local stability (dominant eigenvalue; ${\lambda }_{{\max }}$) of the R–N subsystem as ${a}_{{RN}}$ is increased for ${l}_{N} \, > \, {l}_{R}$, ${l}_{N}={l}_{R}$, and ${l}_{N} < {l}_{R}$, where ${l}_{R}$ and ${l}_{N}$ are the rate at which nutrients are lost to the external environment from compartment R and N, respectively. Solid lines are real parts and dashed lines are complex parts of ${\lambda }_{{\max }}$.

As the maximum rate of nutrient uptake (${a}_{{RN}}$) is increased (i.e., R–N interaction strength), stability is generally increased (Fig. 4d), with the real part of the dominant eigenvalue (${\lambda }_{{\max }}$) tending from 0 (i.e., where ${a}_{{RN}}$ allows the resource to persist) towards an asymptote of ${-l}_{R}$ (see Supplementary Results 2C). Numerical analysis reveals that the asymptote at ${-l}_{R}$ can be approached from above or below depending on the relative leakiness of the R and N compartments (i.e., the rate at which nutrients are lost to the external environment from compartment R (${l}_{R}$) and N (${l}_{N}$)). For ${l}_{N} \, > \, {l}_{R}$ (Fig. 4d), the R–N module only follows a monotonic return to equilibrium as interaction strength is increased, with increased interaction strength only tending to increased stability (i.e., reduce return time). For ${l}_{N} < {l}_{R}$ (Fig. 4d), the R–N module follows a monotonic return to equilibrium for weak (Fig. 4a) and strong (Fig. 4c) interaction strength, but modest overshoot dynamics are observed for intermediate interaction strength (Fig. 4b). Stability tended to increase with interaction strength for weak to intermediate interaction strength (i.e., dominant eigenvalue becomes more negative), then slightly decrease as interaction strength became strong. A special case exists when ${l}_{R}={l}_{N}$ (Fig. 4d), where stability increases with interaction strength until ${\lambda }_{{\max }}$ becomes locked in at ${-l}_{R}$, indicating stability does not change regardless of any further increase in interaction strength. Overall, the R–N interaction tends to generally stabilize in all cases (dominant eigenvalue goes from zero to a more negative saturating value with monotonic dynamics), although there are some intermediate cases that produce complex eigenvalues that suggest population dynamic overshoot potential (Fig. 4b). Note that we obtain qualitatively similar results when implicitly strengthening the R–N interaction by increasing nutrient loading (see Supplementary Results 2D and Supplementary Fig. 1). Now, given the above framework for coupled C–R modules—where weak C–R interactions with underlying monotonic dynamics dampen the oscillatory potential of strong C–R interactions—the underlying monotonic dynamics of the R–N module suggest that R–N interactions ought to be stabilizing when coupled to strong C–R interactions. Further, the underlying increase in stability (i.e., more rapid return to equilibrium) as R–N interaction strength is increased suggests the stabilizing potential of the R–N module ought to increase as the interaction becomes stronger.

**Fig. 5:**
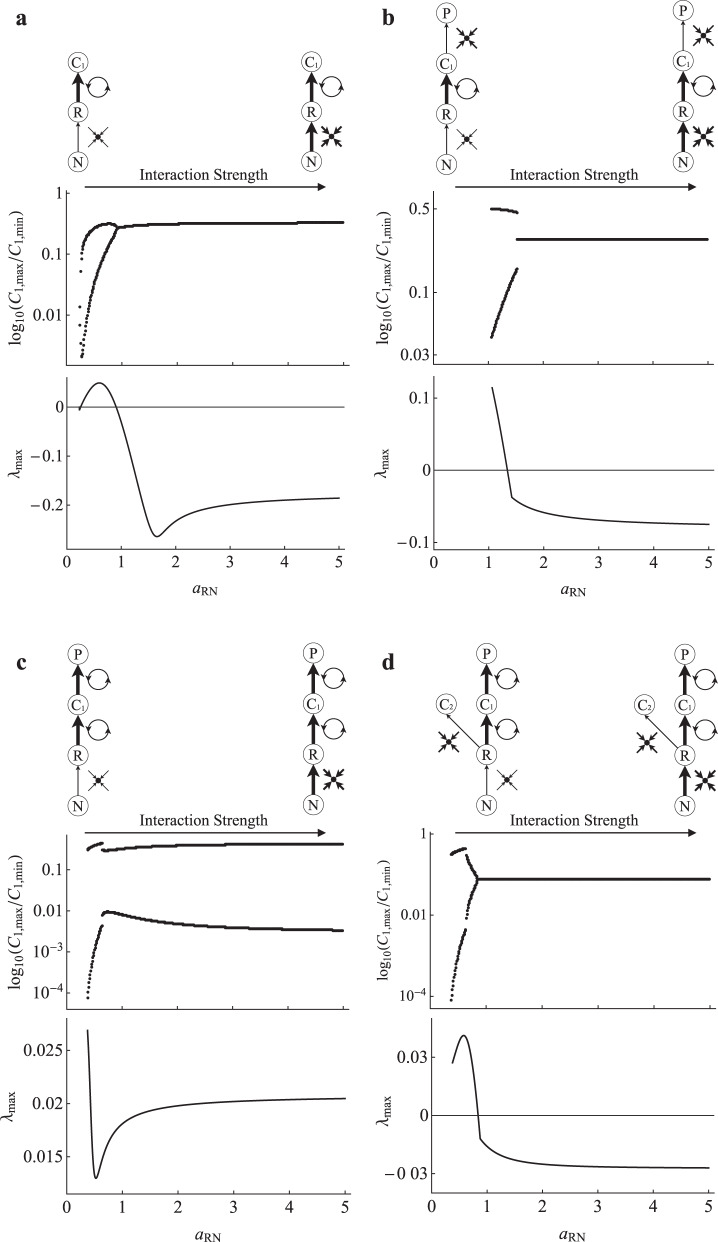
Nutrient-limited food chain stability. **a–d** Non-equilibrium dynamics (log_10_(*C*_1,max_/*C*_1,min_)) and equilibrium stability (real part of the dominant eigenvalue; ${\lambda }_{{\max }}$) of the C–R–N, P–C–R–N with a single oscillator, P–C–R–N with coupled oscillators, and P–C_1_–C_2_–R–N modules, respectively, as ${a}_{{RN}}$ is varied.

**Fig. 6:**
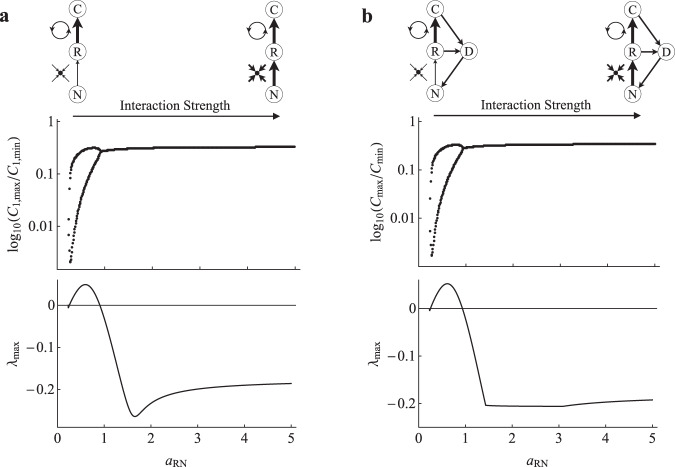
Nutrient-limited ecosystem module stability. **a**, **b** Non-equilibrium dynamics (log_10_(*C*_max_/*C*_min_)) and equilibrium stability (real part of the dominant eigenvalue; ${\lambda }_{{\max }}$) of the C–R–N nutrient-limited food chain model and the C–R–N–D nutrient-limited ecosystem model, respectively, as ${a}_{{RN}}$ is varied.

To look into this conjecture, we first coupled R–N to multiple configurations of strong and expectantly oscillatory C–R interactions and increased R–N interaction strength (${a}_{{RN}}$). Following this, we added nutrient cycling and repeated the experiment to demonstrate that our results can be generalized to nutrient-limited ecosystem models. The full equations and parameter values for each model are listed in Supplementary Results 3A–D and 4A, B. We begin with the C–R–N system, where C–R and R–N are coupled through R (Fig. 5a). The initial increase in ${a}_{{RN}}$ implicitly strengthens the C–R interaction and fuels the oscillatory potential of C–R and cycles emerge almost immediately after C is able to persist. As ${a}_{{RN}}$ is increased further the cycles disappear and we obverse a steep stabilization phase, followed by a modest period of destabilization. Adding a weakly coupled predator gives a similar outcome, although the system continually stabilizes as ${a}_{{RN}}$ is increased (Fig. 5b). If the P–C interaction is strengthened (i.e., both C_1_–R and P–C_1_ are strong, the recipe for chaos), R–N is unable to dampen oscillations even with a strong interaction strength, although a strong interaction gives tighter bound cycles than a weak interaction (Fig. 5c). We next add a weakly coupled consumer to the nutrient-limited food chain with strong P–C_1_ and C_1_–R interactions (Fig. 5d). As seen previously, this interaction draws energy out of the strong pathway, partially muting oscillatory potential. Thus, the ability for a strong R–N interaction to once again return the system to a stable equilibrium is not surprising. Finally, we add a detrital compartment to show that strong R–N interactions remain potent stabilizers in the context of nutrient cycling (Fig. 6b) when compared to a nutrient-limited food chain without nutrient cycling (Fig. 6a).

Note that we repeat our analysis of higher order modules by implicitly increasing R–N interaction strength through nutrient loading (see Supplementary Results 3E and 4C and Supplementary Figs. 2 and 3). In all cases, increased nutrient loading led to less stable dynamics, consistent with DeAngelis’ (1992) paradox of enrichment finding where increased nutrient loading lead to destabilizing autotroph–herbivore oscillations.

## Discussion

Here, we have revisited modular theory to show that in contrast to C–R generated theory, the R–N module is inherently stable, with strong R–N interactions acting as potent stabilizers in higher order systems. However, under intermediate interaction strengths the R–N module did display overshoot dynamic potential (i.e., dominant eigenvalue has complex parts; Fig. 4d). This overshoot potential has been known to excite instability in higher order modules^16^. This was likely captured in the higher order modules where we observed brief periods of destabilization at weak to intermediate interactions strengths before the system was stabilized as the R–N interaction became strong. Further, maximal stability of both the underlying R–N module and the higher order module can occur at more intermediate interaction strengths (e.g., Fig. 5a, c). Importantly though, the region that follows maximal stability remains more stable than the dynamics observed for weak R–N interactions and thus overall, strong R–N interactions tend to act as potent stabilizers when coupled to higher order food web modules.

Interestingly, R–N interactions tend to act as a potent stabilizer despite the fact that increases in interaction strength cascades through the system to increase the overall flux of nutrients through C–R interactions—something known to be destabilizing in food web theory^16^. Consider the C–R–N model under equilibrium conditions. In turning up the R–N interaction strength, N shrinks in size as nutrients are extracted at a higher rate with C growing because of the top-down control it exerts on R. Thus, the flow of nutrients through C–R is implicitly increased in a manner similar to the familiar paradox of enrichment^21^. C–R theory would then predict the system to become increasingly unstable^2^, but as we have observed here (Fig. 5), this is not the case. Despite implicit increases to the strength of C–R interactions, strong monotonic R–N interactions appear to be such potent stabilizers that they are able to overwhelm the potential instability invoked in C–R by the increased flux of nutrients as R–N interaction strength is increased.

The degree to which R–N interactions can stabilize a system (i.e., eliminate oscillatory dynamics), however, seems to be limited by the overall potential instability in C–R interactions. Recall the relative stability of the different food web modules without R–N. In the case of a food chain, a single oscillatory C–R interaction produces well bounded limit cycles (a more stable dynamical outcome; Fig. 3b), coupled oscillatory C–R interactions produce chaos (a less stable dynamical outcome; Fig. 3c), and the addition of a weak pathway brings chaotic dynamics back to bounded cycles (a more stable dynamical outcome relative to the coupled oscillators; Fig. 3d). Then, when coupled to R–N, increasing R–N interaction strength dampens out oscillations entirely in the single oscillator food chain (Fig. 5b), only bring cycles to a tighter bound in the coupled oscillator food chain (Fig. 5c), and entirely dampen oscillations with the addition of a weak pathway (Fig. 5d). The corollary is that the structure-stability foundation of C–R generated theory can be predictably extended to include R–N interactions.

Despite strong R–N interactions acting as potent stabilizers in higher order systems, implicit increases to R–N interaction strength through nutrient loading tend to result in the paradox of enrichment. This can be reconciled in the following way. First note that R–N interaction strength refers specifically to the rate of maximum uptake by R (i.e., ${a}_{{RN}}$) rather than the general sense of any increase in flux through the interaction^22^. Now this appears to be in line with what DeAngelis originally put forward, at least implicitly, for the R–N subsystem. Consider first that DeAngelis (1992) recreated the classic paradox of enrichment with a simple four compartment ecosystem model, finding that high nutrient loading led to destabilizing autotroph–herbivore oscillations. He also noted, however, that when a system is nutrient-limited, sufficiently strong reactive decreases to irruptions in autotroph biomass by the limiting-nutrient compartment can dampen an oscillatory C–R system. In other words, how much R “feels” the decline it imposes on N can create a self-damping effect. This aligns with what we have found here where strong R–N interactions can dampen whole system dynamics while heightened nutrient loading leads to the paradox of enrichment. Therefore, we posit that increasing nutrient loading implicitly strengthens and fuels the oscillatory potential of C–R interactions such that it overwhelms the system, whereas for any given level of nutrient loading increasing the maximum rate of nutrient uptake by R pulls the R–N module back towards a state of strong reactive decreases in the limiting-nutrient pool and thus acts to dampen destabilizing C–R oscillations.

Mathematically, differences in dynamical outcomes as the uptake terms are increased is found in the modelling of basal compartments. Consider the three compartment, two interaction, non-recycling, P–C–R and C–R–N systems. All interactions are modelled with a type II functional response (see Supplementary Results equations (1.3) and (3.0); note the Monod equation is mathematically identical to a type II functional response) and the top two trophic levels have linear loss terms. Nonetheless, increasing the basal interaction term is stabilizing in C–R–N (Fig. 5a) and destabilizing in P–C–R (Fig. 3b). The difference in dynamics therefore arises from N being modelled with density-independent growth (${I}_{N}$) and a linear loss term ($-{l}_{N}N$) while the R in P–C–R is modelled with density-dependent growth (${rR}$) and a non-linear self-damping loss term ($-r{R}^{2}/K$) (i.e., logistic growth). We conjecture this difference between the dynamical properties of R in P–C–R and N in C–R–N creates a scenario where despite an implicit time lag, N is unable to escape the top-down forces of R and overshoot its equilibrium density following a perturbation (i.e., dynamics remain largely out-of-phase between R and N to create a self-damping effect). This stabilizing force then increases as the interaction becomes stronger and greater top-down control is exerted on N by R. This is in contrast to an implicit lag allowing R to escape the top-down control of C and overshoot its equilibrium, eventually creating a limit cycle as the interaction is further strengthened^5^. Nonetheless, more work—such as the change in correlation between R and N dynamics under non-equilibrium conditions as interaction strength is increased (sensu Rooney et al.^10^)—is needed to fully elucidate the mechanism underlying the unique dynamical outcome observed here.

The modular framework employed here operates such that complex systems are reduced to modules that can be studied and then be slowly pieced back together into an understanding of whole system dynamics. Modules themselves exist at different levels of complexity, as we have seen here. The C–R module was used to understand higher order food web modules, which has been coherently scaled back up to whole food webs^4,6^. Analogously, we examined the stabilizing properties of the R–N module to understand its ability to confer stability in a fully formed nutrient-cycling ecosystem model. It remains to extend these findings to the scale of whole ecosystems, an important area of future work. Furthermore, it is equally important to extend our findings to the empirical realm. Experimental aquatic ecosystem microcosms employed by Fussmann et al.^23^ to show the paradox of enrichment perfectly match the model outlined here, providing a reasonable first step toward testing these ideas in real systems.

Taken altogether, nutrient-limited and energetic approaches to theory offer distinct perspectives into the stabilizing mechanisms of food webs in space and time. Nonetheless, the goal of a nutrient-limited ecosystem approach is not to replace the energetic models of food web theory, but rather to integrate the concepts of food web theory (specifically food web structure and dynamics) with the inevitable influence of material cycling processes and limiting nutrients that have dominated ecosystem theory. Nutrient-limited models capture, in a sense, a specific brand of limited resource growth, as opposed to the all-encompassing intrinsic growth rate (i.e., $r$; see Supplementary Results) of energetic models. They ask, all else equal, how limiting nutrients can alter the dynamics, stability, and ultimately persistence of food webs. Nutrient-limited ecosystem models attempt to give insight into one of many factors that limit the growth of resources in space and time, with the ultimate goal of synthesizing this insight with other factors to form an integrative theory. As pointed out by O’Neil et al.^13^, ecosystems are inevitably studied from limited viewpoints and it is imperative to maintain a holistic view of ecological theory as insight is gained through these limited viewpoints.

### Reporting summary

Further information on research design is available in the Nature Research Reporting Summary linked to this article.

## Supplementary information

Supplementary Information

Reporting Summary

## Data Availability

There were no data used in this study and thus no data are available.
