# Supplementary material for: Strong nutrient-plant interactions enhance the stability of ecosystems
Source: Commun Biol. 2021 Oct 20;4:1202. doi: 10.1038/s42003-021-02737-3 (PMC8528884; doi:10.1038/s42003-021-02737-3)
Supplement: Supplementary file 1 — Supplementary Information [file 42003_2021_2737_MOESM1_ESM.pdf]

Supplementary Information

Supplementary Results

1. Construction and Analysis of Community Models

A. Basic C-R Module

For the analysis of the basic C-R module, we use a classic Rosenzweig-MacArthur C-R system, with type II functional responses and logistic resource growth. The specific equations we use are as follows:

$$\frac{dR}{dt} = rR\left(1 - \frac{R}{K}\right) - \frac{a_{CR}CR}{R + R_0}$$
$$\frac{dC}{dt} = \frac{ea_{CR}CR}{R + R_0} - m_C C \tag{1.0}$$

where R is resource biomass and C is consumer biomass. For the logistic growth of the resource, we have intrinsic growth rate r and carrying capacity K. To model the interactions between the species we have the attack rate of the consumer on the resource a_{CR}, the assimilation rate e, and half-saturation density R_0. The mortality rate of the consumer is m_C.

Time series (Fig. 2) were conducted over 300 integration steps, with the resource initialized at R^* while the consumer was initialized at $\frac{1}{2}C^*$. Parameters: $r = 1$, $K = 2$, $R_0 = 0.4$, $e = 0.5$, $m_C = 0.5$, with a_{CR} varied as follows: $a_{CR} = 1.25$ (Fig. 2a), $a_{CR} = 1.47$ (Fig. 2b), and $a_{CR} = 1.60$ (Fig. 2c).

The local stability analyses for the model was based around the interior equilibrium:

$$R^* = \frac{R_0 m_C}{ea_{CR} - m_C}$$
$$C^* = \frac{r}{a_{CR}}\left(1 - \frac{R^*}{K}\right)(R^* + R_0) \tag{1.1}$$

Stability was determined by first taking the partial derivatives about the interior equilibrium, giving the Jacobian matrix:

$$\begin{bmatrix} -\frac{rR^*}{K} + \frac{rR^*}{R^* + R_0}\left(1 - \frac{R^*}{R^* + R_0}\right) & -\frac{a_{CR}R^*}{R^* + R_0}\left(1 - \frac{R^*}{R^* + R_0}\right) \\ \frac{a_{CR}C^*}{R^* + R_0}\left(1 - \frac{R^*}{R^* + R_0}\right) & 0 \end{bmatrix} = A \qquad (1.2)$$

from which the equation for the eigenvalues were given by the determinant $\det(A - I\lambda) = 0$. Fig. 2d shows λ_{max} as a_{CR} is varied from 1.2 to 1.8. Parameters: $r = 1$, $K = 2$, $R_0 = 0.4$, $e = 0.5$, $m_C = 0.5$.

B. P-C-R Food Chain Model

We extended the basic C-R module to a three-compartment food chain model, adding a top predator with a type II functional response. The specific equations were as follows:

$$\frac{dR}{dt} = rR\left(1 - \frac{R}{K}\right) - \frac{a_{CR}CR}{R + R_0}$$

$$\frac{dC}{dt} = \frac{ea_{CR}CR}{R + R_0} - m_C C - \frac{a_{PC}PC}{C + C_0} \qquad (1.3)$$

$$\frac{dP}{dt} = \frac{ea_{PC}PC}{C + C_0} - m_P P$$

where R is resource biomass, C is consumer biomass, and P is predator biomass. For the logistic growth of the resource, we have intrinsic growth rate r and carrying capacity K. To model the interaction between species we have two attack rates: consumer on the resource a_{CR} and predator on the consumer a_{PC}, with an assimilation rate e, and two half-saturation densities: the consumer on the resource R_0 and the predator on the consumer C_0. Finally, we have two mortality rates: the consumer m_C, the predator m_P.

Time series (Fig. 3a-c) were plotted after 9000 integration time steps, ensuring steady state, upwards to 10 000 integration time steps. Parameters: $r = 1$, $K = 2$, $R_0 = 0.5$, $C_0 = 0.3$, $e = 0.5$,

$m_C = 0.5$, $m_P = 0.3$ with a_{CR} and a_{PC} varied as follows: $a_{CR} = 1.3$ and $a_{PC} = 1.25$ (Fig. 3a), $a_{CR} = 2$ and $a_{PC} = 1.25$ (Fig. 3b), and $a_{CR} = 2$ and $a_{PC} = 3$ (Fig. 3c). Initial conditions for the resource, consumer, and predator were $\frac{1}{2}R^*$, C^*, and P^*, where each is the numerically calculated equilibrium density.

C. P-C1-C2-R Specialist Model

We extended the P-C-R food chain model to a four-compartment model, adding a second consumer with a type II functional response. The specific equations were as follows:

$$\begin{aligned}
\frac{dR}{dt} &= rR\left(1 - \frac{R}{K}\right) - \frac{a_{C_1R}C_1R}{R + R_0} - \frac{a_{C_2R}C_2R}{R + R_0} \\
\frac{dC_1}{dt} &= \frac{ea_{C_1R}C_1R}{R + R_0} - m_{C_1}C_1 - \frac{a_{PC_1}PC_1}{C_1 + C_0} \\
\frac{dC_2}{dt} &= \frac{ea_{C_2R}C_2R}{R + R_0} - m_{C_2}C_2 \\
\frac{dP}{dt} &= \frac{ea_{PC_1}PC_1}{C_1 + C_0} - m_P P
\end{aligned} \tag{1.4}$$

where R is resource biomass, C_1 is consumer 1 biomass, C_2 is consumer 2 biomass and P is predator biomass. For the logistic growth of the resource, we have intrinsic growth rate r and carrying capacity K. To model the interaction between species we have three attack rates: consumer 1 on the resource a_{C_1R}, consumer 2 on the resource a_{C_2R} and predator on consumer 1 a_{PC_1}, with an assimilation rate e, and two half-saturation densities: consumer i on the resource R_0 and the predator on consumer i C_0. Finally, we have three mortality rates: consumer i m_{C_i} and the predator m_P.

Time series (Fig. 3d) was plotted after 9000 integration time steps, ensuring steady state, upwards to 10 000 integration time steps. Parameters: $r = 1$, $K = 2$, $R_0 = 0.5$, $C_0 = 0.5$, $e = 0.5$,

$m_C = 0.5$, $m_P = 0.3$, $a_{C_1R} = 2$, $a_{C_2R} = 1.55$, and $a_{PC_1} = 3$. Initial conditions for the resource, consumer 1, consumer 2, and predator were $\frac{1}{2}R^*$, ${C_1}^*$, ${C_2}^*$, and P^*, where each is the numerically calculated equilibrium density.

D. P-C1-C2-R Generalist Model

We extended the P-C1-C2-R model to a diamond model, having a predator with a multi-species functional response. The specific equations were as follows:

$$\begin{aligned}
\frac{dR}{dt} &= rR\left(1-\frac{R}{K}\right) - \frac{a_{C_1R}C_1R}{R+R_0} - \frac{a_{C_2R}C_2R}{R+R_0} \\
\frac{dC_1}{dt} &= \frac{ea_{C_1R}C_1R}{R+R_0} - m_{C_1}C_1 - \frac{a_{PC_1}PC_1}{1+a_{PC_1}h_{PC_1}C_1 + a_{PC_2}h_{PC_2}C_2} \\
\frac{dC_2}{dt} &= \frac{ea_{C_2R}C_2R}{R+R_0} - m_{C_2}C_2 - \frac{a_{PC_2}PC_2}{1+a_{PC_1}h_{PC_1}C_1 + a_{PC_2}h_{PC_2}C_2} \\
\frac{dP}{dt} &= \frac{eP(a_{PC_1}C_1 + a_{PC_2}C_2)}{1+a_{PC_1}h_{PC_1}C_1 + a_{PC_2}h_{PC_2}C_2} - m_PP
\end{aligned} \tag{1.5}$$

where R is resource biomass, C_1 is consumer 1 biomass, C_2 is consumer 2 biomass and P is predator biomass. For the logistic growth of the resource, we have intrinsic growth rate r and carrying capacity K. To model the interaction between species we have four attack rates: consumer i on the resource a_{C_iR} and the predator on consumer i a_{PC_i}, with an assimilation rate e, a handling time of consumer i by the predator h_{PC_i} and a half-saturation density of consumer i on the resource R_0. Finally, we have three mortality rates: consumer i m_{C_i} and the predator m_P.

Time series (Fig. 3e) was plotted after 9000 integration time steps, ensuring steady state, upwards to 10 000 integration time steps. Parameters: $r = 1$, $K = 2$, $R_0 = 0.5$, $e = 0.8$, $m_C = 0.3$, $m_P = 0.3$, $h_{PC_1} = 0.1$, $h_{PC_2} = 0.1$, $a_{C_1R} = 2$, $a_{C_1R} = 0.6$, $a_{PC_1} = 3$, $a_{PC_2} = 0.2$. Initial

conditions for the resource, consumer 1, consumer 2, and predator were $\frac{1}{2}R^*$, ${C_1}^*$, ${C_2}^*$, and P^*, where each is the numerically calculated equilibrium density.

2. Construction and Analysis of the R-N Module

A. R-N Module

For the analysis of the R-N module, we use a limiting-nutrient pool and resource that are open to the environment, with Monod-like nutrient uptake by the resource. The specific equations we use are as follows:

$$\begin{aligned} \frac{dN}{dt} &= I_N - \frac{a_{RN}NR}{k+N} - l_N N \\ \frac{dR}{dt} &= \frac{a_{RN}NR}{k+N} - l_R R \end{aligned} \tag{2.00}$$

where N is limiting-nutrient pool nutrients and R is nutrients assimilated by the resource. The limiting-nutrient pool has external inputs I_N. To model the uptake of nutrients we have the maximum rate of nutrient uptake by the resource a_{RN} and a half saturation density of k. Nutrients are lost from compartment i according to l_i.

B. Local Stability Analyses

The local stability analyses for the model was based around the interior equilibrium:

$$\begin{aligned} R^* &= \frac{(I_N - l_N N^*)(k+N^*)}{a_{RN}N^*} \\ N^* &= \frac{k\, l_R}{a_{RN} - l_R} \end{aligned} \tag{2.01}$$

We impose biological feasibility on our system with the following restrictions on parameters: (1) parameter values must be positive and (2) parameterizations must confer positive abundances of R^* and N^*, such that the following inequalities are satisfied:

$$a_{RN} > l_R \tag{2.02}$$

$$I_N(a_{RN} - l_R) > l_N\ l_R k \tag{2.03}$$

With this restriction, we now consider stability criteria outlined in Strogatz (2018) to determine local stability (i.e., the sign of the real parts of the eigenvalues) about the interior equilibrium. We first linearize the system about the interior equilibrium, giving the Jacobian matrix:

$$\begin{bmatrix} -\frac{I_N(l_R - a_{RN})^2 + l_N {l_R}^2 k}{k\ l_R\ a_{RN}} & -l_R \\ \frac{(l_R - a_{RN})(I_N(l_R - a_{RN}) + l_N l_R k)}{k\ l_R\ a_{RN}} & 0 \end{bmatrix} = A \tag{2.04}$$

The trace of matrix A is given by:

$$\tau = -\frac{I_N(l_R - a_{RN})^2 + l_N {l_R}^2 k}{k\ l_R\ a_{RN}} \tag{2.05}$$

which satisfies the inequality $\tau < 0$ for all biologically feasible parameterizations.

The determinant of matrix A is given by:

$$\det(A) = \frac{(l_R - a_{RN})(I_N(l_R - a_{RN}) + l_N l_R k)}{k\ a_{RN}} \tag{2.06}$$

which can be shown to satisfy the inequality $\det(A) > 0$ as follows:

$\det(A) > 0$ is equal to

$$\frac{(l_R - a_{RN})(I_N(l_R - a_{RN}) + l_N l_R k)}{k\ a_{RN}} > 0 \tag{2.07}$$

Given the inequality $a_{RN} > l_R$, $(l_R - a_{RN})$ must be negative, and the inequality $\det(A) > 0$ can be satisfied if

$$I_N(l_R - a_{RN}) + l_N l_R k < 0 \tag{2.08}$$

which rearranges to:

$$I_N(a_{RN} - l_R) > l_N l_R k \tag{2.09}$$

which has been satisfied by our restriction for biological feasibility (2.03).

Therefore, given that the trace and determinant of the Jacobian for the interior equilibrium satisfy the criteria $\tau < 0$ and $\det(A) > 0$ for biologically feasible parametrizations, we can conclude that the dominant eigenvalue has real parts that are only negative and that the R-N system is locally stable.

C. Interaction Strength and Local Stability

We determine how local stability (i.e., the magnitude of the dominant eigenvalue) changes as a_{RN} is increased (Fig. 4). Again, we restrict our parameters for biologically feasibility as outlined in the previous section, satisfying inequalities (2.02) and (2.03). As a_{RN} is increased from the point at which the resource can persist, three qualitatively distinct patterns of stabilization occur depending on which of the following inequalities/equality are satisfied: $l_N > l_R$, $l_N = l_R$, $l_N < l_R$ (Fig. 4). We are able to prove the case of $l_N = l_R$ as general with the following proof.

We first expand the determinant $\det(A - I\lambda) = 0$ which yields the characteristic equation:

$$\lambda^2 + \lambda \frac{I_N(l_R - a_{RN})^2 + l_N {l_R}^2 k}{k\, l_R\, a_{RN}} + \frac{(l_R - a_{RN})(I_N(l_R - a_{RN}) + l_N l_R k)}{k\, a_{RN}} = 0 \qquad (2.10)$$

with the solutions to $\lambda_{1,2}$ given by:

$$\lambda_{1,2} = \frac{-\frac{I_N(l_R - a_{RN})^2 + l_N {l_R}^2 k}{k\, l_R\, a_{RN}} \pm \sqrt{\left(\frac{I_N(l_R - a_{RN})^2 + l_N {l_R}^2 k}{k\, l_R\, a_{RN}}\right)^2 - 4\,\frac{(l_R - a_{RN})(I_N(l_R - a_{RN}) + l_N l_R k)}{k\, a_{RN}}}}{2} \qquad (2.11)$$

Substituting l_N and l_R for l_i in equation (2.11) and rearranging gives:

$$\lambda_{1,2} = \frac{-\frac{I_N(l_i - a_{RN})^2 + {l_i}^3 k}{k\, l_i\, a_{RN}} \pm \sqrt{\left(\frac{I_N(l_i - a_{RN})^2 + {l_i}^2 k(l_i - 2a_{RN})}{k\, l_i\, a_{RN}}\right)^2}}{2} \qquad (2.12)$$

simplifying to

$$\lambda_1 = -l_i \tag{2.13}$$

$$\lambda_2 = -\frac{2I_N(l_i - a_{RN})^2 - {l_i}^2 k(l_i - 2a_{RN}) - {l_i}^3 k}{2k\ l_i\ a_{RN}}$$

where λ_1 evaluates the positive root and λ_2 evaluates the negative root. Evaluating λ_2 as a_{RN} approaches ∞ yields:

$$\lim_{a_{RN}\to\infty} \lambda_2 = -\infty \tag{2.14}$$

Therefore, λ_{max} must descend from zero until $\lambda_2 = \lambda_1$, at which point $\lambda_{max} = -l_R$ for all increasing values of a_{RN}.

For the general case of $l_N \neq l_R$, we are able to analytically show where λ_{max} tends to for very large values of a_{RN}. Rearranging equation (2.11) gives:

$$\lambda_{1,2} = \frac{C_1 {a_{RN}}^2 + C_2 a_{RN} + C_3 \pm \sqrt{C_4 {a_{RN}}^4 + C_5 {a_{RN}}^3 + C_6 {a_{RN}}^2 + C_7 a_{RN} + C_8}}{2\ k\ l_R\ a_{RN}} \tag{2.15}$$

where

$$C_1 = -I_N$$

$$C_2 = 2I_N l_R$$

$$C_3 = -I_N {l_R}^2 - l_N {l_R}^2 k$$

$$C_4 = {I_N}^2$$

$$C_5 = -4{I_N}^2 l_R - 4I_N {l_R}^2 k$$

$$C_6 = 6{I_N}^2 {l_R}^2 + 8l_N {l_R}^3 k + 4l_N {l_R}^3 k^2 + 2I_N l_N {l_R}^2 k$$

$$C_7 = -4{I_N}^2 {l_R}^3 - 4I_N l_N {l_R}^3 k + l_N {l_R}^4 k^2 + I_N {l_R}^4 k$$

$$C_8 = {I_N}^2 {l_R}^4 + {l_N}^2 {l_R}^4 k^2 + 2I_N l_N {l_R}^4 k$$

Given a positive coefficient for the highest order term under the square root sign, λ_{max} must be real for very large values of a_{RN}. Rationalizing the numerator of equation (2.15) and evaluating the limit of $\lambda_{1,2}$ as a_{RN} approaches ∞ yields:

$$\lim_{a_{RN}\to\infty} \lambda_1 = -l_R$$
$$\lim_{a_{RN}\to\infty} \lambda_2 = -\infty \qquad (2.16)$$

where λ_1 evaluates the positive root and λ_2 evaluates the negative root. Therefore, λ_{max} must tend from 0 to $-l_R$ as a_{RN} is increased.

Numerical analysis reveals that the horizontal asymptote can be approached from below or above, with λ_{max} approaching from above for $l_N > l_R$ and from below for $l_N < l_R$ (Fig. 4). Parameters for Fig. 4 are as follows:

For the case of $l_N > l_R$: $I_N = 0.1$, $k = 0.5$, $l_N = 0.2$, $l_R = 0.1$.

For the case of $l_N = l_R$: $I_N = 0.1$, $k = 0.5$, $l_N = 0.2$, $l_R = 0.2$.

For the case of $l_N < l_R$: $I_N = 0.1$, $k = 0.5$, $l_N = 0.2$, $l_R = 0.3$.

To determine the generality of each result we perform the experiment of increasing a_{RN} 100 times, randomizing the parameters within uniform ranges. For the case of $l_N < l_R$, we hold $l_N = 0.1$ constant, randomizing other parameters in the following ranges: $I_N = Uniform(0.01,1)$, $l_R = Uniform(0.11,1)$, $k = Uniform(0.1,1)$. In all cases, λ_{max} descends below $-l_R$, before approaching $-l_R$ from below. For the case of $l_N > l_R$, we hold $l_R = 0.1$ constant, randomizing other parameters in the following ranges: $I_N = Uniform(0.01,1)$, $l_N = Uniform(0.11,1)$, $k = Uniform(0.1,1)$. In all cases, λ_{max} approaches $-l_R$ from above.

Last, we consider the generality of zero and non-zero complex parts for λ_{max} when $l_N > l_R$ and $l_N < l_R$. For the case of $l_N < l_R$, we hold $l_N = 0.1$ constant, randomizing other parameters in the following ranges: $I_N = Uniform(0.01,1)$, $l_R = Uniform(0.11,1)$, $k = Uniform(0.1,1)$.

In all cases, λ_{max} has non-zero complex parts for decreasing values and only real parts as λ_{max} subsequently increases toward $-l_R$, consistent with the qualitative pattern shown in Fig. 4. For the case of $l_N > l_R$, we hold $l_R = 0.1$ constant, randomizing other parameters in the following ranges: $I_N = Uniform(0.01,1)$, $l_N = Uniform(0.11,1)$, $k = Uniform(0.1,1)$. In all cases, λ_{max} has only real parts.

D. Nutrient Loading and Local Stability

We extended the analysis of interaction strength and stability by implicitly strengthening the R-N interaction through nutrient loading (i.e., forcing I_N) and asking how stability changes.

Starting with the special case of $l_N = l_R$, equation (2.12) gives the following:

$$\lambda_1 = -l_i$$
$$\lim_{I_N \to \infty} \lambda_2 = -\infty \tag{2.17}$$

where λ_1 evaluates the positive root and λ_2 evaluates the negative root of equation (2.12). Therefore, λ_{max} must descend from zero until $\lambda_2 = \lambda_1$, at which point $\lambda_{max} = -l_i$ for all increasing values of I_N.

For the general case of $l_N \neq l_R$, we are able to analytically show where λ_{max} tends to for very large values of I_N. Given only positive values can be obtained for the highest order I_N term under the square root sign of equation (2.11), λ_{max} must be real for very large values of I_N. Rationalizing the numerator of equation (2.11) and evaluating the limit of $\lambda_{1,2}$ as I_N approaches ∞ yields:

$$\lim_{I_N \to \infty} \lambda_1 = -l_R$$
$$\lim_{I_N \to \infty} \lambda_2 = -\infty \tag{2.18}$$

where λ_1 evaluates the positive root and λ_2 evaluates the negative root. Therefore, λ_{max} must tend from 0 to $-l_R$ as I_N is increased.

Numerical analysis reveals that the horizontal asymptote can be approached from below or above, with λ_{max} approaching from above for $l_N > l_R$ and from below for $l_N < l_R$ (Supplementary Fig. 1). Parameters for Supplementary Fig. 1 are as follows:

For the case of $l_N > l_R$: $a_{RN} = 0.8$, $k = 0.5$, $l_N = 0.2$, $l_R = 0.1$.

For the case of $l_N = l_R$: $a_{RN} = 0.8$, $k = 0.5$, $l_N = 0.2$, $l_R = 0.2$.

For the case of $l_N < l_R$: $a_{RN} = 0.8$, $k = 0.5$, $l_N = 0.2$, $l_R = 0.3$.

To determine the generality of each result we perform the experiment of increasing I_N 100 times, randomizing the parameters within uniform ranges. For the case of $l_N < l_R$, we hold $l_N = 0.1$ constant, randomizing other parameters in the following ranges: $a_{RN} = Uniform(0.1,3)$, $l_R = Uniform(0.11,1)$, $k = Uniform(0.1,1)$. In all cases, λ_{max} descends below $-l_R$, before approaching $-l_R$ from below. For the case of $l_N > l_R$, we hold $l_R = 0.1$ constant, randomizing other parameters in the following ranges: $a_{RN} = Uniform(0.1,3)$, $l_N = Uniform(0.11,1)$, $k = Uniform(0.1,1)$. In all cases, λ_{max} approaches $-l_R$ from above.

Last, we consider the generality of zero and non-zero complex parts for λ_{max} when $l_N > l_R$ and $l_N < l_R$. For the case of $l_N < l_R$, we hold $l_N = 0.1$ constant, randomizing other parameters in the following ranges: $a_{RN} = Uniform(0.1,3)$, $l_R = Uniform(0.11,1)$, $k = Uniform(0.1,1)$. In all cases, λ_{max} has non-zero complex parts for decreasing values and only real parts as λ_{max} subsequently increases toward $-l_R$, consistent with the qualitative pattern shown in Fig. 4. For the case of $l_N > l_R$, I hold $l_R = 0.1$ constant, randomizing other parameters in the following ranges: $a_{RN} = Uniform(0.1,3)$, $l_N = Uniform(0.11,1)$, $k = Uniform(0.1,1)$. In all cases, λ_{max} has only real parts.

3. Construction and Analysis of Nutrient-Limited Food Chains

A. C-R-N Model

We extend the basic R-N module to a three-compartment nutrient-limited model, adding a consumer with a type II functional response. The specific equations are as follows:

$$\frac{dN}{dt} = I_N - \frac{a_{RN}RN}{k+N} - l_N N$$
$$\frac{dR}{dt} = \frac{a_{RN}RN}{k+N} - l_R R - \frac{a_{RC}CR}{R+R_0} \qquad (3.0)$$
$$\frac{dC}{dt} = \frac{ea_{RC}CR}{R+R_0} - l_C C$$

where N is limiting-nutrient pool nutrients, R is nutrients assimilated by the resource, and C is nutrients assimilated by the consumer. The limiting-nutrient pool has external inputs I_N. To model the uptake of nutrients we have two maximum uptake rates: uptake from the limiting-nutrient pool by the resource a_{RN} and uptake from resource by the consumer a_{CR}, with an assimilation efficiency e, and two half saturation densities: the resource on the limiting nutrient pool k and the consumer on the resource R_0. Nutrients are lost from compartment i according to l_i.

B. P-C-R-N Model

We extended the C-R-N model to a four-compartment nutrient-limited model, adding a top predator with a type II functional response. The specific equations are as follows:

$$\frac{dN}{dt} = I_N - \frac{a_{RN}RN}{k+N} - l_N N$$
$$\frac{dR}{dt} = \frac{a_{RN}RN}{k+N} - l_R R - \frac{a_{CR}CR}{R+R_0} \qquad (3.1)$$
$$\frac{dC}{dt} = \frac{ea_{CR}CR}{R+R_0} - l_C C - \frac{a_{PC}PC}{C+C_0}$$

$$\frac{dP}{dt} = \frac{ea_{PC}PC}{C + C_0} - l_P P$$

where N is limiting-nutrient pool nutrients, R is nutrients assimilated by the resource, C is nutrients assimilated by the consumer, and P is nutrients assimilated by the predator. The limiting-nutrient pool has external inputs I_N. To model the uptake of nutrients we have three maximum uptake rates: uptake from the limiting-nutrient pool by the resource a_{RN}, uptake from resource by the consumer a_{CR}, and uptake from consumer by the predator a_{PC}, with an assimilation efficiency e, and three half saturation densities: the resource on the limiting nutrient pool k, the consumer on the resource R_0, and the predator on the consumer C_0. Nutrients are lost from compartment i according to l_i.

C. P-C_1-C_2-R-N Model

We extended the P-C-R-N model to a five-compartment nutrient-limited model, adding a second consumer with a type II functional response. The specific equations are as follows:

$$\frac{dN}{dt} = I_N - \frac{a_{RN}RN}{k + N} - l_N N$$

$$\frac{dR}{dt} = \frac{a_{RN}RN}{k + N} - l_R R - \frac{a_{C_1R}C_1R}{R + R_0} - \frac{a_{C_2R}C_2R}{R + R_0}$$

$$\frac{dC_1}{dt} = \frac{ea_{C_1R}C_1R}{R + R_0} - l_{C_1}C_1 - \frac{a_{PC_1}PC_1}{C_1 + C_0} \tag{3.2}$$

$$\frac{dC_2}{dt} = \frac{ea_{C_2R}C_2R}{R + R_0} - l_{C_2}C_2$$

$$\frac{dP}{dt} = \frac{ea_{PC_1}PC_1}{C_1 + C_0} - l_P P$$

where N is limiting-nutrient pool nutrients, R is nutrients assimilated by the resource, C_1 is nutrients assimilated by the first consumer, C_2 is nutrients assimilated by the second consumer and P is nutrients assimilated by the predator. The limiting-nutrient pool has external inputs I_N. To model the uptake of nutrients we have four maximum uptake rates: uptake from the limiting-

nutrient pool by the resource a_{RN}, uptake from resource by the first consumer a_{C_1R}, uptake from resource by the second consumer a_{C_2R}, and uptake from the first consumer by the predator a_{PC_1}, with an assimilation efficiency e, and three half saturation densities: the resource on the limiting nutrient pool k, consumer i on the resource R_0, and the predator on the first consumer C_0. Nutrients are lost from compartment i according to l_i.

D. Stability Analysis

Global stability was determined over a range of values for a_{RN} (Fig. 5). The lower endpoint of the range for a_{RN} is defined as the point where all compartments persist at densities greater than zero, with the upper endpoint for a_{RN} set to 5. The analyses were run for 10 000 integration steps, with the local maxima and minima of the consumer density collected over the last 100 integration steps. The analyses were only performed on the last 100 integration steps to ensure steady state.

The local stability of the interior equilibrium was analyzed over a range of values for a_{RN} identical to global stability analyses (Fig. 5). The interior equilibrium, Jacobian matrix, and eigenvalues were calculated numerically using Mathematica 11.

The parameter values for Fig. 5 are as follows:

(a) $I_N = 0.5$, $l_N = 0.1$, $l_R = 0.2$, $l_C = 0.5$, $k = 0.5$, $R_0 = 0.5$, $e = 0.5$, $a_{CR} = 1.6$.

(b) $I_N = 0.5$, $l_N = 0.1$, $l_R = 0.2$, $l_C = 0.5$, $l_P = 0.3$, $k = 0.5$, $R_0 = 0.5$, $C_0 = 0.3$, $e = 0.5$, $a_{CR} = 2$, $a_{CP} = 1.25$.

(c) $I_N = 0.5$, $l_N = 0.1$, $l_R = 0.2$, $l_C = 0.5$, $l_P = 0.3$, $k = 0.5$, $R_0 = 0.5$, $C_0 = 0.3$, $e = 0.5$, $a_{CR} = 2$, $a_{CP} = 3$.

(d) $I_N = 0.5$, $l_N = 0.1$, $l_R = 0.2$, $l_{C_1} = 0.5$, $l_{C_2} = 0.5$, $l_P = 0.3$, $k = 0.5$, $R_0 = 0.5$, $C_0 = 0.3$, $e = 0.5$, $a_{C_1R} = 2$, $a_{C_2R} = 1.55$ $a_{CP} = 3$.

To determine the generality of the stability relationship between the R-N and C-R module we perform the experiment of increasing a_{RN} 100 times for the C-R-N system. We randomized all parameters within uniform ranges and checked for feasibility across the range of a_{RN} values (0.1-10). The upper limit of the range was increased from 5 to 10 as changes to the parameterization of the system are relative and can shift the quantitative points of stabilization. Thus, a wider range of tested values allows us to confirm that a transition to increasing stability (as observed in Fig. 5a where an initial increase is destabilizing, before the system is stabilized) that is shifted right is still consistent with the qualitative pattern of stabilization (i.e., as the interaction strength is increased, any instability that initially emerges is dampened by stronger interaction strengths). We repeated the randomization process until 100 feasible parameter sets were obtained. We then performed the experiment for each parameter set, using visual confirmation to ensure that the qualitative pattern described in Fig. 5 (i.e., strong R-N interactions correspond to cycles of decreased amplitude or a stable equilibrium is reached, when compared to weak R-N interactions) remained for all cases. Note that a_{CR} was held constant at the same value, as the point of the experiment is to determine the ability of a strong R-N interaction to dampen out the oscillatory potential of a strong C-R interaction. Parameters were randomized within the range of the following distributions:

(a) $I_N = Uniform(0.4, 0.6)$, $l_N = Uniform(0.01, 0.2)$, $l_R = Uniform(0.1, 0.3)$, $l_C = Uniform(0.1, 0.3)$, $k = Uniform(0.4, 0.6)$, $R_0 = Uniform(0.4, 0.6)$, $e = Uniform(0.7, 0.9)$, $a_{CR} = 1.6$.

In all cases, strong R-N interactions corresponded to cycles of decreased amplitude when compared to weak R-N interactions, or cycles were damped out to a stable equilibrium as interaction strength was increased.

E. Nutrient Loading Stability Analysis

Global stability was determined over a range of values for I_N (Supplementary Fig. 2). The lower endpoint of the range for I_N is defined as the point where all compartments persist at densities greater than zero, with the upper endpoint for I_N set to 3. The analyses were run for 10 000 integration steps, with the local maxima and minima of the consumer density collected over the last 100 integration steps. The analyses were only performed on the last 100 integration steps to ensure steady state.

The local stability of the interior equilibrium was analyzed over a range of values for I_N identical to global stability analyses. The interior equilibrium, Jacobian matrix, and eigenvalues were calculated numerically using Mathematica 11.

The parameter values for Supplementary Fig. 2 are as follows:

(a) $l_N = 0.1$, $l_R = 0.2$, $l_C = 0.5$, $k = 0.5$, $R_0 = 0.5$, $e = 0.5$, $a_{RN} = 0.8$, $a_{CR} = 1.6$.

(b) $l_N = 0.1$, $l_R = 0.2$, $l_C = 0.5$, $l_P = 0.3$, $k = 0.5$, $R_0 = 0.5$, $C_0 = 0.3$, $e = 0.5$, $a_{RN} = 0.8$, $a_{CR} = 2$, $a_{CP} = 1.25$.

(c) $l_N = 0.1$, $l_R = 0.2$, $l_C = 0.5$, $l_P = 0.3$, $k = 0.5$, $R_0 = 0.5$, $C_0 = 0.3$, $e = 0.5$, $a_{RN} = 0.8$, $a_{CR} = 2$, $a_{CP} = 3$.

(d) $l_N = 0.1$, $l_R = 0.2$, $l_{C_1} = 0.5$, $l_{C_2} = 0.5$, $l_P = 0.3$, $k = 0.5$, $R_0 = 0.5$, $C_0 = 0.3$, $e = 0.5$, $a_{RN} = 0.8$, $a_{C_1R} = 2$, $a_{C_2R} = 1.55$ $a_{CP} = 3$.

4. Construction and Analysis of Nutrient-limited Ecosystem Models

A. C-R-N-D Model

We extended the C-R-N model to a four-compartment nutrient-limited ecosystem model, adding a detrital compartment that mineralizes nutrients back to the limiting-nutrient pool. The specific equations are as follows:

$$\frac{dN}{dt} = I_N + d_D D - \frac{a_{RN} R N}{k + N} - l_N N$$

$$\frac{dR}{dt} = \frac{a_{RN}RN}{k+N} - l_R R - m_R R - \frac{a_{CR}CR}{R+R_0} \tag{4.0}$$

$$\frac{dC}{dt} = \frac{ea_{CR}CR}{R+R_0} - l_C C - m_C C$$

$$\frac{dD}{dt} = m_C C + m_R R + \frac{(1-e)a_{CR}CR}{R+R_0} - d_D D - l_D D$$

where N is limiting-nutrient pool nutrients, R is nutrients assimilated by the resource, C is nutrients assimilated by the consumer, and D is nutrients present in detritus. The limiting-nutrient pool has an external input I_N and an internal recycling input $d_D D$ where d_D is the rate of mineralization of nutrients in the detrital compartment. To model the uptake of nutrients we have two maximum uptake rates: uptake from the limiting-nutrient pool by the resource a_{RN} and uptake from resource by the consumer a_{CR}, with an assimilation efficiency e, and two half saturation densities: the resource on the limiting nutrient pool k and the consumer on the resource R_0. Nutrients are lost from compartment i according to l_i. Nutrients from compartment C and R are conserved in the system and contribute to detritus according to the mortality term m_i. Inefficiencies in nutrient assimilation and sloppy feeding by consumers also contribute towards the detrital pool.

B. Stability Analysis

Global stability was determined over a range of values for a_{RN} (Fig. 6). The lower endpoint of the range for a_{RN} is defined as the point where all compartments persist at densities greater than zero, with the upper endpoint for a_{RN} set to 5. The analyses were run for 10 000 integration steps, with the local maxima and minima of the consumer density collected over the last 100 integration steps. The analyses were only performed on the last 100 integration steps to ensure steady state.

The local stability of the interior equilibrium was analyzed over a range of values for a_{RN} identical to global stability analyses. The interior equilibrium, Jacobian matrix, and eigenvalues were calculated numerically using Mathematica 11.

The parameter values for Fig. 6 are as follows:

(a) $I_N = 0.5$, $l_N = 0.1$, $l_R = 0.2$, $l_C = 0.5$, $k = 0.5$, $R_0 = 0.5$, $e = 0.5$, $a_{CR} = 1.6$.

(b) $I_N = 0.5$, $l_N = 0.1$, $l_R = 0.1$, $m_R = 0.1$, $l_C = 0.3$, $m_C = 0.2$, $l_D = 0.2$, $d_D = 0.01$, $k = 0.5$, $R_0 = 0.5$, $e = 0.5$, $a_{CR} = 1.6$.

C. Nutrient Loading Stability Analysis

Global stability was determined over a range of values for I_N (Supplementary Fig. 3). The lower endpoint of the range for I_N is defined as the point where all compartments persist at densities greater than zero, with the upper endpoint for I_N set to 3. The analyses were run for 10 000 integration steps, with the local maxima and minima of the consumer density collected over the last 100 integration steps. The analyses were only performed on the last 100 integration steps to ensure steady state.

The local stability of the interior equilibrium was analyzed over a range of values for I_N identical to global stability analyses. The interior equilibrium, Jacobian matrix, and eigenvalues were calculated numerically using Mathematica 11.

The parameter values for Supplementary Fig. 3 are as follows:

(a) $l_N = 0.1$, $l_R = 0.2$, $l_C = 0.5$, $k = 0.5$, $R_0 = 0.5$, $e = 0.5$, $a_{RN} = 0.8$, $a_{CR} = 1.6$.

(b) $l_N = 0.1$, $l_R = 0.1$, $m_R = 0.1$, $l_C = 0.3$, $m_C = 0.2$, $l_D = 0.2$, $d_D = 0.01$, $k = 0.5$, $R_0 = 0.5$, $e = 0.5$, $a_{RN} = 0.8$, $a_{CR} = 1.6$.

Supplementary Figures

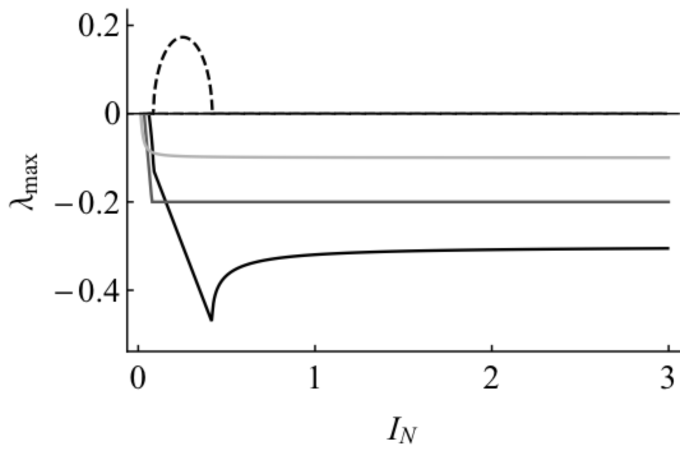

Supplementary Figure 1. R-N Stability response to increasing nutrient loading. Local stability (dominant eigenvalue; λ_{max}) of the R-N subsystem as I_N is increased for $l_N > l_R$ (light gray), $l_N = l_R$ (dark gray), and $l_N < l_R$ (black line). Solid lines are real parts and dashed lines are complex parts of λ_{max}.

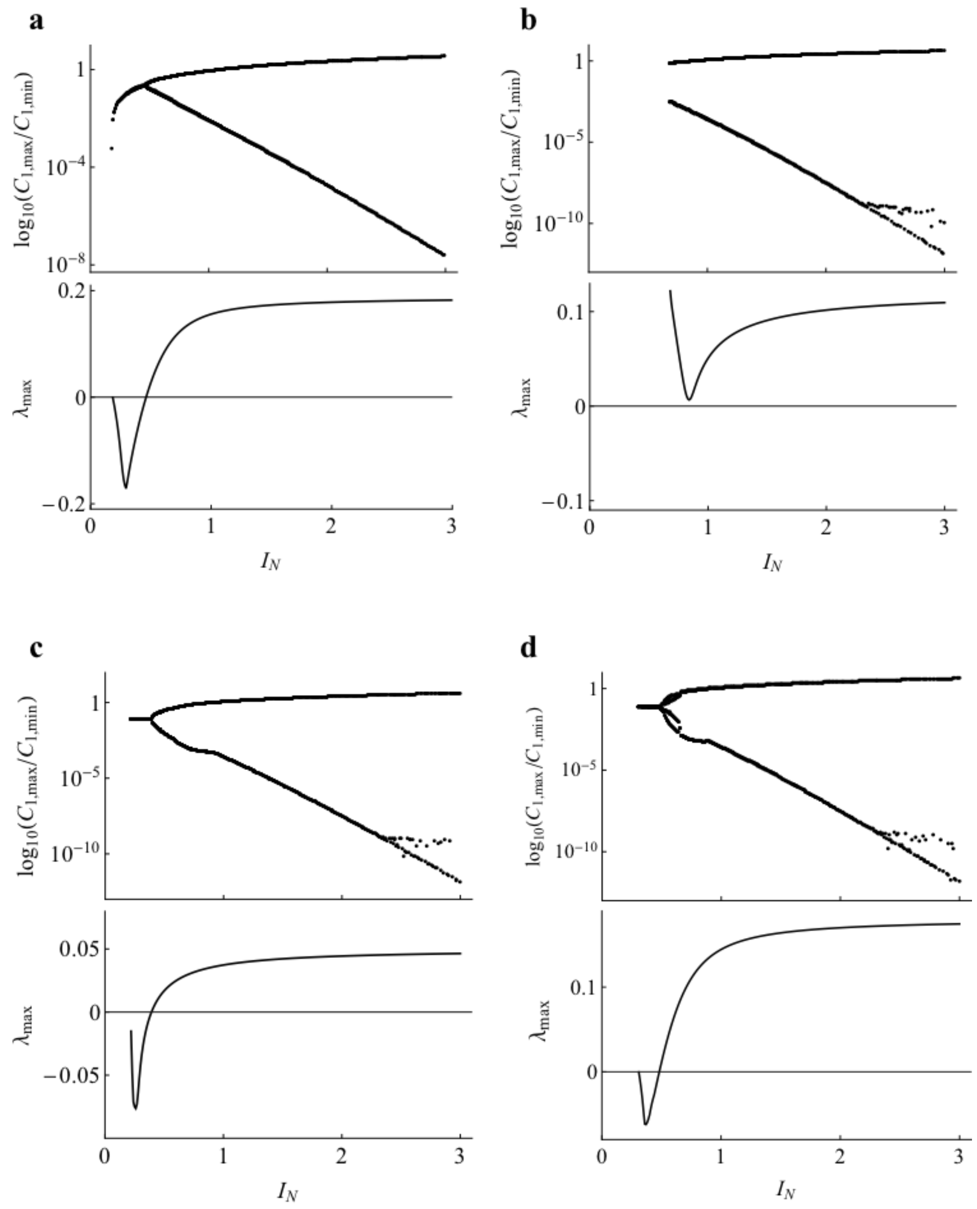

Supplementary Figure 2. Nutrient-limited food chain stability in response to nutrient loading. a, b, c, d, Non-equilibrium dynamics ($\log_{10}(C_{1,max}/C_{1,min})$) and equilibrium stability (dominant eigenvalue; λ_{max}) of the C-R-N, P-C-R-N with a single oscillator, P-C-R-N with coupled oscillators, and P-C_1-C_2-R-N modules, respectively, as nutrient loading (I_N) is varied.

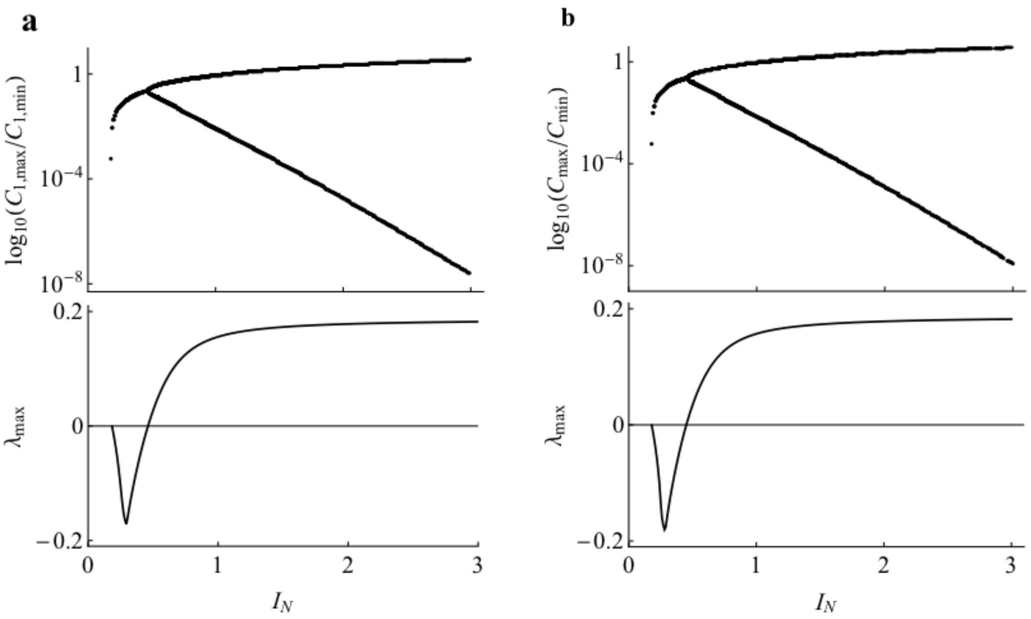

Supplementary Figure 3. Nutrient-limited ecosystem module stability in response to nutrient loading. a, b, Non-equilibrium dynamics ($\log_{10}(C_{1,max}/C_{1,min})$) and equilibrium stability (dominant eigenvalue; λ_{max}) of the C-R-N and C-R-N-D models, respectively, as nutrient loading (I_N) is varied.
